# Supplementary material for: Spatio-temporal machine learning for multi-horizon prediction of bluetongue outbreaks
Source: PLoS Comput Biol. 2026 Oct 21;22(10):e1014870. doi: 10.1371/journal.pcbi.1014870 (PMC13750650; doi:10.1371/journal.pcbi.1014870)
Supplement: Supplementary file 1 [file pcbi.1014870.s003.docx]

**Response to Reviewers**

We thank the Editor and the reviewers for handling and carefully reviewing the manuscript and for providing constructive comments and suggestions that have improved the quality of this work. All comments and suggestions have been carefully considered and addressed throughout the manuscript.

Line numbers refer to the tracked-changes version of the manuscript. Reviewer comments are shown in bold. Where text has been modified, the revised wording is provided below each comment in *red italics*.

Reviewer #1: Your machine learning model is mathematically very strong. However, it oversimplifies the biology of the Bluetongue virus (BTV). By ignoring key virological factors, the model turns a complex disease into a "black box," making it difficult to use for real-world epidemiology.

**We thank the reviewer for these thoughtful comments. Predictive models necessarily involve simplifications of complex biological systems, and we agree that the scope of these simplifications should be clearly defined. To address the concerns raised, we expanded the biological description of the dataset, conducted additional sensitivity analyses evaluating host-species and serotype information, clarified the representation of climatic predictors, and revised the Discussion to more clearly define the scope and limitations of the modelling framework.**

Here are a few points that the authors may wish to address in a revision:

1.BTV behaves completely differently in cows (who act as silent carriers) than in sheep (who get severely sick and die). Your data shows France's cases are mostly cows, while Greece's are mostly sheep. Grouping them all together into one generic data point hides how the virus actually spreads from silent carriers to vulnerable herds.

**Response: We thank the reviewer for this important comment, and we would like to clarify the scope of the additional analysis, as our original wording was not sufficiently explicit about what was and was not done. To address this concern, we revised the Methods to define the host and serotype indicators explicitly, expanded the dataset description to quantify host composition across countries, and revised the Discussion accordingly (Methods, Lines 577–588).**

**We agree that cattle and small ruminants differ in their epidemiological roles in bluetongue transmission. To be explicit about what the additional analysis did: we did not re-define the prediction target by host species. The outcome remained unit-level outbreak occurrence. Instead, each reporting unit–month was annotated with two binary indicators derived from the same WAHIS records — one indicating that at least one outbreak in that unit and month involved cattle, and one indicating that at least one involved sheep or goats — and these indicators entered the model only at lags of one to six months, within the same six-month look-back window used for all other predictors. These indicators are now defined explicitly in the Methods (Lines 577-588).**
**We also examined whether a host-stratified outcome would be informative at this spatial scale, and found that it would not partition the data in the way the aggregated target might suggest. Across 2005–2024, of the unit–months in which an outbreak was reported, 100% in France involved cattle, 96.9% in Greece involved sheep or goats, and in Italy 90.8% involved cattle while 55.8% involved sheep or goats, with 46.9% involving both. A cattle-specific outcome for France is therefore identical to the aggregated outcome, and a small-ruminant-specific outcome for Greece is almost identical to it. Host groups co-occur within administrative reporting units rather than separating into distinct ones.**

**Consequently, stratifying the outcome by host species at the administrative unit–month scale would not isolate cattle and small-ruminant transmission: it would largely reproduce the aggregated result in France and Greece, and would split an already sparse outcome in Italy. Incorporating the lagged host indicators had little effect on forecasting performance in France or Italy and improved it only slightly in Greece (Results, Lines 238–244). A genuinely host-specific analysis of transmission from subclinically infected cattle into susceptible small-ruminant flocks would require outbreak- or farm-level modelling rather than the administrative reporting scale used here.**

**As an additional assessment of biological heterogeneity, we conducted a further sensitivity analysis in response to the reviewer’s comments by incorporating lagged serotype indicators. These analyses produced only small changes in forecasting performance across the three countries, with performance improving only in Italy (Results, Lines 238–244).**

**The Discussion has also been revised to acknowledge that, although host species and circulating serotypes differ in their epidemiological characteristics, incorporating host and serotype information provided limited additional predictive information beyond the spatial, temporal, and environmental predictors already included in the forecasting framework (Discussion, Lines 368–370).**

*Lines 238–244 (Results):* ***2.6 Host and serotype sensitivity analyses***

*Incorporating lagged host indicators had little effect on forecasting performance in France or Italy. In Greece, host indicators produced a modest improvement, increasing the mean horizon F₂ score from 0.9881 to 0.9888 and reducing false positives from 64 to 60, while recall remained unchanged. Incorporating lagged serotype indicators produced mixed effects, slightly reducing performance in France and Greece but improving performance in Italy, where the mean horizon F₂ score increased from 0.5729 to 0.5821 and false positives decreased from 175 to 148.*

*Lines 368–370 (Discussion): The limited improvements observed when lagged host and serotype information were incorporated further suggest that these cross-country differences are driven primarily by broader spatial and environmental heterogeneity rather than host composition or circulating serotypes alone.*

*Lines 439–442 (Discussion):* Several potentially important determinants of BTV risk were not included because they are not consistently available across countries, including vaccination coverage, animal movements, vector abundance and species composition, circulating BTV serotypes, and broader ecological or socio-economic factors influencing livestock connectivity.

*Lines 577–588 (Methods): Host and serotype variables were not included in the primary forecasting framework because the prediction target was unit-level outbreak occurrence irrespective of host species or serotype. To assess whether these biological differences influenced forecasting performance, lagged host and serotype indicators were incorporated in separate sensitivity analyses. For the host analysis, each reporting unit–month was annotated with two binary indicators derived from the same WAHIS records: one indicating that at least one outbreak reported in that unit and month involved cattle, and one indicating that at least one involved sheep or goats. These indicators are not mutually exclusive, since a unit–month may contain outbreaks in both host groups. Serotype indicators were constructed analogously, one per major circulating serotype. In both analyses the indicators entered the model only at lags of one to six months, within the same six-month look-back window used for all other predictors, so that no host or serotype information from the forecast target month was available to the model. The prediction target itself was unchanged in these analyses: unit-level outbreak occurrence irrespective of host species or serotype.*

2. The model uses simple "monthly average temperatures," which doesn't reflect how the virus actually grows in midges. Viral replication requires specific heat accumulation. For example, an average temperature of 15°C with hot days and freezing nights affects the virus very differently than a constant 15°C

**Response: We thank the reviewer for this important observation. To address this concern, we revised the Methods to clarify that climate predictors represent monthly summaries averaged across each administrative reporting unit and therefore do not explicitly capture daily thermal accumulation, diurnal temperature variation, or temperature-dependent viral replication within Culicoides vectors (Methods, Lines 619–623). We also clarified that the forecasting framework uses a six-month sliding window of climate predictors rather than only the current month's conditions, with mean, minimum and maximum temperature, temperature range, precipitation, rolling precipitation, humidity, surface pressure and wind speed included for each of the six observed months preceding the forecast horizon (Methods, Lines 671–674). Finally, we revised the Discussion to emphasise that these climate variables should be interpreted as statistical indicators of environmental suitability rather than direct representations of the biological processes governing vector development or viral replication (Discussion, Lines 294–296).**

**We agree that temperature accumulation and daily thermal variation influence vector competence and viral development and are not explicitly represented by monthly climate summaries. The forecasting framework presented here was designed as an operational surveillance model using routinely available monthly environmental data rather than a mechanistic model of vector biology. Although monthly climate summaries cannot fully represent the underlying thermal biology, the inclusion of multiple lagged climate variables enabled the model to capture temporal environmental conditions preceding each forecast. These revisions clarify both the scope of the forecasting framework and the limitations associated with using monthly aggregated climate data.**

*Lines 619–623: Climate predictors therefore represent average monthly conditions for each administrative reporting unit and do not capture fine-scale spatial variation within individual reporting units. Monthly climate summaries were used throughout the forecasting framework; consequently, daily thermal accumulation, diurnal temperature variation, and temperature-dependent viral replication within Culicoides vectors were not explicitly modelled.*

*Lines 294–296: Although monthly climate summaries do not explicitly represent vector development or viral replication processes, they appear to capture sufficient biologically relevant variation to support accurate forecasting at the administrative unit–month scale.*

*Lines 671–674: Thus, climate variables were not represented only by the current month; mean, minimum and maximum temperature, temperature range, precipitation, rolling precipitation, humidity, surface pressure and wind speed were included for each of the six observed months preceding the forecast horizon.*

3.There are over 20 BTV strains (like BTV-4 vs. BTV-8). They each have different levels of severity, spread speeds, and weather sensitivities. The model treats them all as the exact same disease, which skews the predictions

**Response: We thank the reviewer for this important comment. To address this concern, we expanded the Methods to describe the distribution of circulating BTV serotypes across the three study countries, conducted additional sensitivity analyses incorporating lagged serotype indicators, and revised the Discussion to acknowledge epidemiological differences among circulating serotypes.**

**We agree that BTV serotypes can differ in their pathogenicity, transmission dynamics, and environmental responses. To evaluate whether aggregating outbreaks across serotypes obscured useful predictive information, we incorporated lagged serotype indicators based only on information available in previous months, thereby avoiding information leakage. These analyses produced mixed effects across the three countries. Forecasting performance changed only slightly in France and Greece, whereas performance improved in Italy, with the mean horizon F₂ score increasing from 0.572 to 0.582 and false positives decreasing from 175 to 148.**

**These findings indicate that serotype information contributed additional predictive value in Italy but did not materially alter the overall conclusions across the three study countries. The revised manuscript also makes clear that serotypes were aggregated because the primary outcome was administrative unit-level outbreak occurrence, rather than because they were assumed to have identical biological characteristics.**

*Lines 242-244 (Results): Incorporating lagged serotype indicators produced mixed effects, slightly reducing performance in France and Greece but improving performance in Italy, where the mean horizon F₂ score increased from 0.572 to 0.582 and false positives decreased from 175 to 148.*

*Lines 368–370 (Discussion): The limited improvements observed when lagged host and serotype information were incorporated further suggest that these cross-country differences are driven primarily by broader spatial and environmental heterogeneity rather than host composition or circulating serotypes alone.*

*Lines 439–442 (Discussion): Several potentially important determinants of BTV risk were not included because they are not consistently available across countries, including vaccination coverage, animal movements, vector abundance and species composition, circulating BTV serotypes, and broader ecological or socio-economic factors influencing livestock connectivity.*

*Lines 577–588 (Methods): Host and serotype variables were not included in the primary forecasting framework because the prediction target was unit-level outbreak occurrence irrespective of host species or serotype. To assess whether these biological differences influenced forecasting performance, lagged host and serotype indicators from prior observed months were incorporated in separate sensitivity analyses, thereby avoiding information leakage*

*Line 595–597 (Methods): BTV-8 accounted for 86.0% of serotyped records in France, BTV-4 accounted for 86.0% in Greece, and BTV-1 accounted for 64.4% in Italy, followed by BTV-4 (20.6%) and BTV-8 (13.8%).*

Reviewer #2: Overall, this is a careful and well-conducted study that adapts several well studied models and model assembling approaches to a new disease (BTV). While novelty in method, application and findings is limited, it is still a useful paper and will likely improve NTV forecasting.

Major comments:
The discussion is excessively long (and the results very brief). Consider options to describe results in more detail (e.g. country differences) in the results section where statement can be backed up by figures and tables rather than in mire general terms in the discussion.

**We thank the reviewer for this helpful suggestion. The manuscript has been revised to improve the balance between the Results and Discussion. We have added a new Results subsection describing country-specific model performance, supported by a new figure showing the distribution of model performance across France, Greece, and Italy (Lines 215-236). This allows country-level differences to be presented alongside the supporting results rather than being introduced primarily in the Discussion. The Discussion has been revised accordingly to focus on interpreting these findings, particularly the influence of spatial heterogeneity, administrative scale, and reporting structure on predictability, rather than re-describing country-specific observations. We also streamlined the Discussion by reducing repetition and reorganising the Limitations and Future Directions sections to improve clarity.**

*Line 229:* ***2.5 Country- specific model performance***

*Model performance differed substantially across the three countries (Table 1; Figure 2). Greece consistently achieved the highest predictive performance, with most models attaining F₂ scores close to 1.0 and exhibiting relatively narrow performance distributions across evaluation slices. France also demonstrated consistently strong performance, with the leading models achieving F₂ scores around 0.90, although with greater variability than observed in Greece. In contrast, Italy proved the most challenging setting, exhibiting lower overall F₂ scores and wider performance distributions across models and evaluation slices. Despite these differences in absolute performance, ensemble-based methods consistently ranked among the strongest-performing approaches across all three countries, with the Logit-Weighted Ensemble achieving the highest or near-highest performance in each setting.*

*Figure 2. Distribution of model performance (F₂ score) across evaluation slices for Italy (A), Greece (B), and France (C). Violin plots summarise the distribution of F₂ scores obtained by each model across all administrative unit–forecast horizon evaluation slices. The width of each violin represents the relative density of F₂ scores. Boxplots indicate the interquartile range (IQR), with the horizontal line denoting the median and whiskers extending to 1.5 × IQR. White circles indicate the mean F₂ score for each model. Models are ranked within each country according to their mean F₂ score. Greece exhibited consistently high predictive performance across most models, whereas France showed similarly strong but more variable performance. Italy displayed lower overall F₂ scores and greater dispersion, indicating a more challenging forecasting setting and increased variability in model performance across evaluation slices.*

I was surprised not to see lagged climatic variables included (e..g temperature in previous 1,2,3 months, etc) as they are often key determinants of extended time horizon forecasting and may affect the magnitude of the declining predictive performance with increasing time horizon.

**We thank the reviewer for highlighting the importance of antecedent climatic conditions for extended-horizon forecasting. The forecasting framework already incorporated lagged climate information through a six-month sliding-window formulation; however, we recognise that this was not described clearly in the original manuscript.**

**To address this, we have revised Section 5.2.2 of the Methods to explicitly state that all climate variables—including mean, minimum and maximum temperature, precipitation, humidity, surface pressure and wind speed—were represented as monthly observations over the six months preceding the forecast origin, rather than using only current-month climate conditions. Thus, each prediction was informed by six months of antecedent climate information alongside lagged outbreak-history variables.**

**We have also clarified the climate data description (Section 5.1.3) to emphasise that these predictors were monthly summaries and therefore capture broad seasonal environmental conditions rather than daily thermal dynamics. We hope these revisions make the temporal structure of the climate predictors considerably clearer.**

*Lines 619–623: Climate predictors therefore represent average monthly conditions for each administrative reporting unit and do not capture fine-scale spatial variation within individual reporting units. Monthly climate summaries were used throughout the forecasting framework; consequently, daily thermal accumulation, diurnal temperature variation, and temperature-dependent viral replication within Culicoides vectors were not explicitly modelled.*

*Lines 294–296: Although monthly climate summaries do not explicitly represent vector development or viral replication processes, they appear to capture sufficient biologically relevant variation to support accurate forecasting at the administrative unit–month scale.*

*Lines 671–674: Thus, climate variables were not represented only by the current month; mean, minimum and maximum temperature, temperature range, precipitation, rolling precipitation, humidity, surface pressure and wind speed were included for each of the six observed months preceding the forecast horizon.*

Aggregation of high spatial resolution BTV data prior to extraction of climate features creates an imprecision in the climate variables attributed to the data and isn’t necessary given the many high resolution climate and environmental datasets out there now. It would be worth at lease acknowledging this.

**Response: We thank the reviewer for this helpful suggestion. To address this concern, we revised the Discussion to explicitly acknowledge the limitations associated with aggregating climate variables to the administrative-unit level. We now clarify that, although this approach aligns the climate predictors with the operational forecasting target, it may smooth within-unit climatic heterogeneity and misclassify environmental exposure for outbreaks occurring in localized microclimates. We also identify the use of outbreak- or farm-level climate extraction, or livestock-density-weighted environmental summaries prior to aggregation, as an important direction for future work.**

*Lines 428–433:* Because outbreak records were modelled at administrative-unit resolution, climate summaries were also represented at that scale. This aligns predictors with the operational forecasting target, but it can smooth within-unit climatic heterogeneity and misclassify exposure for outbreaks occurring in localized microclimates. Future work should extract climate features at outbreak or farm coordinates, or use livestock-density-weighted environmental summaries, before aggregating to the surveillance decision unit.

“Where this information was available in WAHIS records, suspected or unconfirmed notifications were excluded.” – would be good to know how many records this excluded and whethere they we in any particular areas / times

**Response: We thank the reviewer for raising this point. On re-examining the WAHIS data and the corresponding manuscript text, we found that no records had in fact been excluded on the basis of suspected or unconfirmed status. The original sentence was therefore inaccurate and could cause confusion. We have removed it from the revised manuscript (Line 575–576).**

No statements on code availability- only refers to data- would expect code to be shared in a reusable way on publication in line with the field.

**We thank the reviewer for highlighting this omission. A Code and Data Availability statement has been added. All code required to reproduce the analyses and forecasting framework, together with the processed modelling datasets, is now publicly available at https://github.com/hungdn210/btv-multihorizon-forecasting and permanently archived at Zenodo(https://doi.org/10.5281/zenodo.21830233) under an MIT licence.**

Additional changes not requested by the reviewers

While preparing this revision we identified a discrepancy between Table 4 and the analysis code. Table 4 listed the predictor variables used by the forecasting framework, but three predictors that the model does use were omitted from the table: reported case count, calendar month, and six-month mean temperature. These variables were present in the modelling datasets and were included in the feature representation of the original analysis; the omission was in the description of the predictor set, not in the analysis itself. We have therefore added these three rows to Table 4 and expanded Section 5.1.4 to describe the reported case count predictor explicitly. No models were re-fitted, no data were changed, and all reported results are unchanged.

Section 5.1.4 (added): Reported case count was also included as a predictor to capture the magnitude of recently observed disease activity within each administrative unit. Unlike the binary outbreak-history indicators, this variable provides a continuous measure of reported outbreak intensity and complements the lagged outbreak-presence features.
