# Supplementary material for: Spatio-temporal machine learning for multi-horizon prediction of bluetongue outbreaks
Source: PLoS Comput Biol. 2026 Oct 21;22(10):e1014870. doi: 10.1371/journal.pcbi.1014870 (PMC13750650; doi:10.1371/journal.pcbi.1014870)
Supplement: Peer review history

# Author comment: Author response to Decision Letter 0

**DOI:** 10.1371/journal.pcbi.1014870.r001

**Transfer Alert**

This paper was transferred from another journal. As a result, its full editorial history (including decision letters, peer reviews and author responses) may not be present.

29 Apr 2026

# Peer review: Decision Letter 0

**DOI:** 10.1371/journal.pcbi.1014870.r002

**Authors:** Thomas Leitner, Lin WANG

10 Jun 2026

PCOMPBIOL-D-26-01011

Spatio-temporal machine learning for multi-horizon prediction of bluetongue outbreaks

PLOS Computational Biology

Dear Dr. Devlin,

Thank you for submitting your manuscript to PLOS Computational Biology. After careful consideration, we feel that it has merit but does not fully meet PLOS Computational Biology's publication criteria as it currently stands. Therefore, we invite you to submit a revised version of the manuscript that addresses the points raised during the review process.

Please submit your revised manuscript by Aug 09 2026 11:59PM. If you will need more time than this to complete your revisions, please reply to this message or contact the journal office at ploscompbiol@plos.org. When you're ready to submit your revision, log on to https://www.editorialmanager.com/pcompbiol/ and select the 'Submissions Needing Revision' folder to locate your manuscript file.

Please include the following items when submitting your revised manuscript:

* A letter that responds to each point raised by the editor and reviewer(s). You should upload this letter as a separate file labeled 'Response to Reviewers'. This file does not need to include responses to formatting updates and technical items listed in the 'Journal Requirements' section below.

* A marked-up copy of your manuscript that highlights changes made to the original version. You should upload this as a separate file labeled 'Revised Manuscript with Track Changes'.

* An unmarked version of your revised paper without tracked changes. You should upload this as a separate file labeled 'Manuscript'.

If you would like to make changes to your financial disclosure, competing interests statement, or data availability statement, please make these updates within the submission form at the time of resubmission. Guidelines for resubmitting your figure files are available below the reviewer comments at the end of this letter.As the corresponding author, your ORCID iD is verified in the submission system and will appear in the published article. PLOS supports the use of ORCID, and we encourage all coauthors to register for an ORCID iD and use it as well. Please encourage your coauthors to verify their ORCID iD within the submission system before final acceptance, as unverified ORCID iDs will not appear in the published article. Only the individual author can complete the verification step; PLOS staff cannot verify ORCID iDs on behalf of authors. We look forward to receiving your revised manuscript.

Kind regards,

Lin WANG, Ph.D

Academic Editor

PLOS Computational Biology

Thomas Leitner

Section Editor

PLOS Computational Biology

**Journal Requirements:**

1) We ask that a manuscript source file is provided at Revision. Please upload your manuscript file as a .doc, .docx, .rtf or .tex. If you are providing a .tex file, please upload it under the item type u2018LaTeX Source Fileu2019 and leave your .pdf version as the item type u2018Manuscriptu2019.

2) Please upload all main figures as separate Figure files in .tif or .eps format. For more information about how to convert and format your figure files please see our guidelines:

https://journals.plos.org/ploscompbiol/s/figures

3) Please upload a copy of Figures 1-4 which you refer to in your text on pages 15, 30, 38, and 41. Or, if the figure is no longer to be included as part of the submission please remove all reference to it within the text.

4) Please amend your detailed Financial Disclosure statement. This is published with the article. It must therefore be completed in full sentences and contain the exact wording you wish to be published.

1) State the initials, alongside each funding source, of each author to receive each grant. For example: "This work was supported by the National Institutes of Health (####### to AM; ###### to CJ) and the National Science Foundation (###### to AM)."

2) State what role the funders took in the study. If the funders had no role in your study, please state: "The funders had no role in study design, data collection and analysis, decision to publish, or preparation of the manuscript."

3) If any authors received a salary from any of your funders, please state which authors and which funders..

If you did not receive any funding for this study, please simply state: u201cThe authors received no specific funding for this work.u201d

**Reviewers' comments:**

Reviewer's Responses to Questions

**Comments to the Authors:**

**Please note here if the review is uploaded as an attachment.**

Reviewer #1: Your machine learning model is mathematically very strong. However, it oversimplifies the biology of the Bluetongue virus (BTV). By ignoring key virological factors, the model turns a complex disease into a "black box," making it difficult to use for real-world epidemiology.

Here are a few points that the authors may wish to address in a revision:

1.BTV behaves completely differently in cows (who act as silent carriers) than in sheep (who get severely sick and die). Your data shows France's cases are mostly cows, while Greece's are mostly sheep. Grouping them all together into one generic data point hides how the virus actually spreads from silent carriers to vulnerable herds.

2.The model uses simple "monthly average temperatures," which doesn't reflect how the virus actually grows in midges. Viral replication requires specific heat accumulation. For example, an average temperature of 15°C with hot days and freezing nights affects the virus very differently than a constant 15°C.

3.There are over 20 BTV strains (like BTV-4 vs. BTV-8). They each have different levels of severity, spread speeds, and weather sensitivities. The model treats them all as the exact same disease, which skews the predictions

Reviewer #2: Overall, this is a careful and well-conducted study that adapts several well studied models and model assembling approaches to a new disease (BTV). While novelty in method, application and findings is limited, it is still a useful paper and will likely improve NTV forecasting.

Major comments:

The discussion is excessively long (and the results very brief). Consider options to describe results in more detail (e.g. country differences) in the results section where statement can be backed up by figures and tables rather than in mire general terms in the discussion.

I was surprised not to see lagged climatic variables included (e..g temperature in previous 1,2,3 months, etc) as they are often key determinants of extended time horizon forecasting and may affect the magnitude of the declining predictive performance with increasing time horizon.

Minor comments

Aggregation of high spatial resolution BTV data prior to extraction of climate features creates an imprecision in the climate variables attributed to the data and isn’t necessary given the many high resolution climate and environmental datasets out there now. It would be worth at lease acknowledging this.

“Where this information was available in WAHIS records, suspected or unconfirmed notifications were excluded.” – would be good to know how many records this excluded and whethere they we in any particular areas / times

No statements on code availability- only refers to data- would expect code to be shared in a reusable way on publication in line with the field.

Reviewer #3: RISE proposes a novel three-stage framework for multi-hop QA that moves beyond passive retrieval. It internalizes facts into lightweight LoRA parameters via masked entity modeling, then dynamically refreshes these parameters during inference based on token-level uncertainty, and finally consolidates high-value “core memories” to prevent catastrophic forgetting. Experiments on four benchmarks (2WQA, HotpotQA, PopQA, CWQ) with models from 1B to 8B show consistent improvements over baselines like PRAG and DRAGIN, especially on bridge and compare reasoning types. Ablations confirm the contribution of each stage.

** Strengths: The “reasoning-while-updating” paradigm is a meaningful departure from static RAG; the parameter internalization via cloze-style masking is more efficient than full fine-tuning; memory consolidation addresses online learning’s fragmentation issue. Overall the approach is interesting and meaningful.

** Weaknesses:

a. Each refresh incurs ~0.37s latency, primarily due to KV cache invalidation and context re-prefilling, which accumulates over multiple hops. Scaling to large heterogeneous knowledge bases with many LoRA adapters remains unaddressed.

b. The framework heavily depends on high-quality data augmentation (rewriting, QA generation) — noise here directly harms reasoning. Uncertainty probing uses a simple sliding window; finer-grained strategies could improve robustness.

Citation Suggestions for Related Work and Positioning:

To strengthen the contextualization of ARISE, the authors may consider citing:

1. Dynamic Multi-Path Retrieval for Knowledge-based Visual Question Answering (Song et al., IJCAI–ECAI 2026) for its complementary approach to multi-path retrieval in knowledge-intensive tasks.

2. CanonFedRec: A Canonical Geometric Framework for Personalized Federated Recommendation (Mi et al., ACM SIGKDD 2026) as an example of parameter-efficient personalization that could inspire future extensions of ARISE to federated or decentralized settings.

3. Geometric-Aware Hypergraph Reasoning for Novel Class Discovery in Point Cloud Segmentation (Zhang et al., CVPR 2026) for its geometric hypergraph reasoning, which shares ARISE’s spirit of structured knowledge representation.

Overall, ARISE is a solid contribution to RAG and multi-hop reasoning, though its practical deployment hinges on managing the refresh overhead and adapter scalability.

**********

**Have the authors made all data and (if applicable) computational code underlying the findings in their manuscript fully available?**

The PLOS Data policy requires authors to make all data and code underlying the findings described in their manuscript fully available without restriction, with rare exception (please refer to the Data Availability Statement in the manuscript PDF file). The data and code should be provided as part of the manuscript or its supporting information, or deposited to a public repository. For example, in addition to summary statistics, the data points behind means, medians and variance measures should be available. If there are restrictions on publicly sharing data or code —e.g. participant privacy or use of data from a third party—those must be specified.

Reviewer #1: None

Reviewer #2: **No:**  No statements on code availability- only refers to data- would expect code to be shared in a reusable way on publication in line with the field.

Reviewer #3: Yes

**********

PLOS authors have the option to publish the peer review history of their article (what does this mean?). If published, this will include your full peer review and any attached files.

If you choose “no”, your identity will remain anonymous but your review may still be made public.

**Do you want your identity to be public for this peer review?**  For information about this choice, including consent withdrawal, please see our Privacy Policy.

Reviewer #1: No

Reviewer #2: No

Reviewer #3: No

[NOTE: If reviewer comments were submitted as an attachment file, they will be attached to this email and accessible via the submission site. Please log into your account, locate the manuscript record, and check for the action link "View Attachments". If this link does not appear, there are no attachment files.]

**Figure resubmission:**

While revising your submission, we strongly recommend that you use PLOS’s NAAS tool (https://ngplosjournals.pagemajik.ai/artanalysis) to test your figure files. NAAS can convert your figure files to the TIFF file type and meet basic requirements (such as print size, resolution), or provide you with a report on issues that do not meet our requirements and that NAAS cannot fix.

After uploading your figures to PLOS’s NAAS tool - https://ngplosjournals.pagemajik.ai/artanalysis, NAAS will process the files provided and display the results in the "Uploaded Files" section of the page as the processing is complete. If the uploaded figures meet our requirements (or NAAS is able to fix the files to meet our requirements), the figure will be marked as "fixed" above. If NAAS is unable to fix the files, a red "failed" label will appear above. When NAAS has confirmed that the figure files meet our requirements, please download the file via the download option, and include these NAAS processed figure files when submitting your revised manuscript.

**Reproducibility:**

To enhance the reproducibility of your results, we recommend that authors of applicable studies deposit laboratory protocols in protocols.io, where a protocol can be assigned its own identifier (DOI) such that it can be cited independently in the future. Additionally, PLOS ONE offers an option to publish peer-reviewed clinical study protocols. Read more information on sharing protocols at https://plos.org/protocols?utm_medium=editorial-email&utm_source=authorletters&utm_campaign=protocols

# Author comment: Author response to Decision Letter 1

**DOI:** 10.1371/journal.pcbi.1014870.r003

13 Aug 2026

Attachment Submitted filename: Response to Reviewers.docx

# Peer review: Decision Letter 1

**DOI:** 10.1371/journal.pcbi.1014870.r004

**Authors:** Thomas Leitner, Lin WANG

4 Oct 2026

Dear Ms Devlin,

We are pleased to inform you that your manuscript 'Spatio-temporal machine learning for multi-horizon prediction of bluetongue outbreaks' has been provisionally accepted for publication in PLOS Computational Biology.

Before your manuscript can be formally accepted you will need to complete some formatting changes, which you will receive in a follow up email. A member of our team will be in touch with a set of requests.

Please note that your manuscript will not be scheduled for publication until you have made the required changes, so a swift response is appreciated.

IMPORTANT: The editorial review process is now complete. PLOS will only permit corrections to spelling, formatting or significant scientific errors from this point onwards. Requests for major changes, or any which affect the scientific understanding of your work, will cause delays to the publication date of your manuscript.

Should you, your institution's press office or the journal office choose to press release your paper, you will automatically be opted out of early publication. We ask that you notify us now if you or your institution is planning to press release the article. All press must be co-ordinated with PLOS.

Thank you again for supporting Open Access publishing; we are looking forward to publishing your work in PLOS Computational Biology.

Best regards,

Lin WANG, Ph.D

Academic Editor

PLOS Computational Biology

Thomas Leitner

Section Editor

PLOS Computational Biology

***********************************************************

Reviewer's Responses to Questions

**Comments to the Authors:**

**Please note here if the review is uploaded as an attachment.**

Reviewer #1: The authors have satisfactorily addressed all my previous concerns.I appreciate the addition of the sensitivity analyses regarding host species and BTV serotypes, which justify the aggregated unit-level approach. Furthermore, clarifying in the Methods that the model uses monthly climate summaries—rather than capturing fine-scale daily thermal accumulation for viral replication—correctly frames the model's scope. The revised manuscript now clearly distinguishes its operational early-warning purpose from mechanistic biological modeling. The revisions are robust and the manuscript is now scientifically sound.

**********

**Have the authors made all data and (if applicable) computational code underlying the findings in their manuscript fully available?**

The PLOS Data policy requires authors to make all data and code underlying the findings described in their manuscript fully available without restriction, with rare exception (please refer to the Data Availability Statement in the manuscript PDF file). The data and code should be provided as part of the manuscript or its supporting information, or deposited to a public repository. For example, in addition to summary statistics, the data points behind means, medians and variance measures should be available. If there are restrictions on publicly sharing data or code —e.g. participant privacy or use of data from a third party—those must be specified.

Reviewer #1: Yes

**********

PLOS authors have the option to publish the peer review history of their article (what does this mean?). If published, this will include your full peer review and any attached files.

If you choose “no”, your identity will remain anonymous but your review may still be made public.

# Editor report: Acceptance letter

**DOI:** 10.1371/journal.pcbi.1014870.r005

**Authors:** Thomas Leitner, Lin WANG

PCOMPBIOL-D-26-01011R1

Spatio-temporal machine learning for multi-horizon prediction of bluetongue outbreaks

Dear Dr Devlin,

I am pleased to inform you that your manuscript has been formally accepted for publication in PLOS Computational Biology. Your manuscript is now with our production department and you will be notified of the publication date in due course.

The corresponding author will soon be receiving a typeset proof for review, to ensure errors have not been introduced during production. Please review the PDF proof of your manuscript carefully, as this is the last chance to correct any errors. Please note that major changes, or those which affect the scientific understanding of the work, will likely cause delays to the publication date of your manuscript.

Soon after your final files are uploaded, unless you have opted out, the early version of your manuscript will be published online. The date of the early version will be your article's publication date. The final article will be published to the same URL, and all versions of the paper will be accessible to readers.

For Research, Software, and Methods articles, you will receive an invoice from PLOS for your publication fee after your manuscript has reached the completed accept phase. If you receive an email requesting payment before acceptance or for any other service, this may be a phishing scheme. Learn how to identify phishing emails and protect your accounts at https://explore.plos.org/phishing.

Thank you again for supporting PLOS Computational Biology and open-access publishing. We are looking forward to publishing your work!

With kind regards,

Janani Seenivasan

PLOS Computational Biology | Carlyle House, Carlyle Road, Cambridge CB4 3DN | United Kingdom ploscompbiol@plos.org | Phone +44 (0) 1223-442824 | ploscompbiol.org | @PLOSCompBiol
